# Autonomic and baroreflex regulations in syndromic and non-syndromic aortopathies: a case–control study

**DOI:** 10.3389/fphys.2025.1719383

**Published:** 2026-01-05

**Authors:** Beatrice Cairo, Nathasha Samali Udugampolage, Francesca Gelpi, Vlasta Bari, Paolo Salvi, Miriam Angolani, Jacopo Taurino, Alberto Porta, Alessandro Pini

**Affiliations:** 1 Department of Biomedical Sciences for Health, University of Milan, Milan, Italy; 2 Cardiovascular-Genetic Center, IRCCS Policlinico San Donato, San Donato Milanese, Italy; 3 Department of Cardiothoracic, Vascular Anesthesia and Intensive Care, IRCCS Policlinico San Donato, San Donato Milanese, Italy; 4 Istituto Auxologico Italiano, Cardiology Unit, IRCCS, Milan, Italy

**Keywords:** autonomic nervous system, baroreflex sensitivity, cardiac control, heart rate variability, heritable connective tissue disorders, thoracic aortic aneurysm

## Abstract

Baroreflex regulation is directly influenced by the mechano-sensitive properties of the baroreceptors. The mechanical and dimensional properties of the aorta are affected in patients with thoracic aortic aneurysm (TAA). We hypothesize that the baroreflex sensitivity (BRS) is modified in TAA patients and that these modifications might be different when the TAA group is divided into syndromic (Synd) and non-syndromic (NonSynd) patients. The aim of the study is to evaluate autonomic and baroreflex control in patients with Synd and NonSynd TAAs. We enrolled 80 TAA patients and divided them into Synd (N = 46) and NonSynd (N = 34) groups. The two groups did not differ in either demographic factors or pharmacological therapy. Autonomic function and BRS, assessed from the heart period (HP) and systolic arterial pressure (SAP) variability, were compared to those of age- and gender-matched healthy controls (HCs, N = 28). Analyses were carried out in the low-frequency (LF, 0.04 Hz–0.15 Hz) and high-frequency (HF, 0.15 Hz–0.4 Hz) bands. The Synd and NonSynd subgroups did not show any significant differences in terms of autonomic control or BRS. We observed that, in the LF band, BRS was lower in TAA patients than in HCs during rest in the supine position (REST), while it was similar during active standing (STAND). STAND reduced the power of HP variability in the HF band and BRS in the LF band while increasing the power of SAP in the LF band in both HCs and TAA patients. Since BRS was lower at REST in both Synd and NonSynd TAA groups than in the HC group, we conclude that BRS is affected by either the dimensional or the mechanical properties of the aorta in relation to the pathology.

## Introduction

The autonomic nervous system modulates sinus node activity and peripheral resistances (Bruno et al., 2012), along with the action of specific reflex mechanisms such as the baroreflex (Parati et al., 1988). Under physiological conditions, blood pressure changes at the baroreceptor sites in the arterial walls directly influence the baroreflex by activating mechanosensitive afferent nerve endings located in the carotid sinuses and within the aortic arch (Hainsworth, 2014; Ghitani and Chesler, 2019). This represents the sensory component of the baroreflex response, while the sympathetic and parasympathetic pathways represent the efferent neural components (Reutersberg et al., 2022). Autonomic function and cardiac baroreflex control are usually evaluated via the study of the spontaneous fluctuations of heart period (HP) and systolic arterial pressure (SAP) (Pomeranz et al., 1985; Pagani et al., 1986; Parlow et al., 1995; Cooke et al., 1999; Marchi et al., 2016a; Marchi et al., 2016b; De Maria et al., 2019a). Baroreflex regulation was investigated by assessing the relationship between HP and SAP spontaneous fluctuations (Laude et al., 2004). The cross-spectral method is one of the most widely applied techniques (de Boer et al., 1985; Cooke et al., 1999; Porta et al., 2002; Porta et al., 2013).

Previous works have evaluated autonomic control and baroreflex in patients with abdominal aortic aneurysm following abdominal aortic surgery (Hoyer et al., 2008; Stein et al., 2001; Reutersberg et al., 2022) or focusing specifically on patients with aneurysm at the level of the ascending aorta (Compostella et al., 2014; Compostella et al., 2015). Chronic changes in baroreflex control are presumed in the population with aortic aneurysms and dissection (Reutersberg et al., 2022) because the aortic arch’s vascular mechanics significantly influence the sensitivity of the cardiac arm of the baroreflex (Klassen et al., 2016). Conversely, evidence is limited in patients with thoracic aortic aneurysm (TAA). TAAs are defined by the site and degree of aortic involvement: most TAAs arise in the aortic root or ascending aorta (60%), followed by the descending aorta, the aortic arch, and, finally, the thoracic-abdominal aorta (Isselbacher, 2005). Manifestations of TAA are often asymptomatic at diagnosis (95% of the patients); for this reason, the identification of complications, including aortic dissection or rupture, is often delayed, which consequently increases mortality rates and cases of underdiagnosed diseases (Faiza and Sharman, 2025). Degenerative etiology is typical in the elderly population aged above 65 years with or without the presence of cardiovascular risk factors, whereas in the population younger than 65 years old, pathological factors contributing to aneurysm formation are significantly associated with family history, the presence of *de novo* genetic mutations, or mutations inherited in an autosomal pattern or X chromosome (Ehlers and Todd, 2017).

Syndromic TAA (Synd) patients have a genetic etiology with multi-organ manifestations: Marfan syndrome (MFS), Loeys–Dietz syndrome (LDS), and vascular Ehlers–Danlos syndrome (vEDS) (Loeys et al., 2006) are the most prevalent. In the case of non-syndromic TAA (NonSynd) patients, the manifestations are limited to the aorta without involvement of other organs and tissues and include familial TAA and bicuspid aortic valve (BAV) syndrome. Genetic mutations associated with Synd patients, with the fibrillin-1 (FBN1) gene typically expressed in MFS and the heterozygous pathogenic variant in COL3A1, represent the diagnostic criterion for vEDS (Robinson et al., 2006; Faivre et al., 2007). At diagnosis, contrast-enhanced computerized tomography scans represent the standard instrumental modality largely used for detecting TAA.

Timely identification of autonomic nervous system and baroreflex dysfunctions is essential for recognizing situations that might increase cardiovascular risk (De Ferrari and Schwartz, 1994), especially in pathological states in which arterial pressure (AP) management is of critical importance (Isselbacher, 2005). We hypothesize that TAA patients may have impaired autonomic and baroreflex controls because enlargement of the aorta might have modified the sensory component of the baroreflex. In addition, the grade of impairment might differ between Synd and NonSynd patients because genetic factors may have influenced neural central integration and efferent pathways beyond the sensory component.

Thus, the aim of this study is to assess autonomic control and baroreflex function through HP and SAP variability in a group of Synd patients compared to NonSynd patients and to healthy control (HC) subjects during a postural challenge known to evoke sympathetic activation, vagal withdrawal, and baroreflex unloading, namely, active standing (STAND) (Steptoe and Vögele, 1990; Catai et al., 2014; Milan-Mattos et al., 2018).

## Materials and methods

### Population characteristics

A single-center cross-sectional study was carried out at the Cardiovascular Genetic Center, IRCCS Policlinico San Donato, San Donato Milanese, Italy. We enrolled 46 Synd and 34 NonSynd patients with TAA between November 2021 and December 2023. The study was conducted in accordance with the Declaration of Helsinki and approved by the Institutional Ethics Committee of Istituto di Ricovero e Cura a Carattere Scientifico (IRCCS) San Raffaele Hospital (protocol code: 54, date of approval: 25-01-2023, ClinicalTrial.gov identifier: NCT05703893 and protocol code: 32/int/2020, date of approval: 04-03-2020, ClinicalTrial.gov identifier: NCT05706532). Written informed consent was obtained from all the subjects involved in the study. Synd patients were identified among those who met the reference criteria for MFS, LDS, and vEDS (Loeys et al., 2010; MacCarrick et al., 2014; Malfait et al., 2017). NonSynd patients were identified among those being followed at the Cardiovascular Genetic Center who did not present extra-aortic manifestations and among subjects with TAA of unknown etiology (e.g., familial TAA) at the time of enrollment, regardless of genetic testing results. The systemic score for Marfan syndrome was computed according to Marelli et al. (2023). The Synd and NonSynd cohorts were compared to HC subjects. For the HC cohort, 28 volunteers were recruited between March 2024 and July 2024. The HC cohort did not present any chronic disease and was not undergoing any pharmacological therapy.

### Inclusion criteria

For both Synd and NonSynd groups, the inclusion criteria were age between 18 and 65 years, TAA diagnosis, and regular follow-up at the Cardiovascular Genetic Center. All patients were identified among those without a history of surgical aortic replacement, who reported spontaneous cardiac sinus rhythm in medical records, and who signed the informed consent forms for the study. Two dimensional (2D)-transthoracic echocardiography (Philips Affiniti 70 G, PureWave; Philips Co, Best, Netherlands) was performed as a standard clinical procedure to evaluate the aortic root and proximal ascending aorta, confirm the diagnosis using a full ultrasound system, and clinically monitor disease progression. Regular therapeutic intake of angiotensin receptor blockers (ARBs) and beta blockers (BBs) was not an exclusion criterion despite their influence on the cardiovascular system as these therapies are widely used in the management and reduction of the rate of progressive aortic root enlargement (Pitcher et al., 2022).

For HCs, the inclusion criteria were age between 18 and 65 years and a spontaneous cardiac sinus rhythm. Volunteers without any history of hypertension, clinically recognized cardiovascular disease, or neurological pathology and not receiving any pharmacological therapy that could affect the cardiovascular or autonomic nervous system were selected. HCs also underwent 2D-transthoracic echocardiography with the same device as the TAA patients to exclude any sign of TAA.

### 2D-transthoracic echocardiographic measurements

Aortic diameter measurements were conducted in accordance with established guidelines (Baumgartner et al., 2010) and obtained in the parasternal long-axis view. Specifically, the aortic valve annulus diameter was measured at the hinge points of the leaflets, the aortic root was measured at the largest diameter within the sinuses of Valsalva, the sinotubular junction diameter was measured at the transition point from the sinus to the tubular aorta, and the ascending aorta diameter was measured at the level of the right pulmonary artery. All echocardiographic images were digitally acquired and analyzed by a single observer.

### Signal acquisition and experimental session

Electrocardiogram (ECG) from lead II using a bioamplifier (BioAmp FE132, ADInstruments, Australia), noninvasive AP from the middle finger via volume-clamp photoplethysmography (CNAP Monitor 500, CNSystems, Austria), and respiratory movement (RESP) signal using a thoracic piezoelectric belt (ADInstruments, Australia) were acquired while resting in supine position (REST) and during STAND. All the subjects were instrumented and instructed to lie down on the same ambulatory bed for 10 min before the start of recording. The acquisition of the REST phase lasted for 10 min after the period of acclimation to the setup and position. Following the REST phase, participants were asked to first sit down and then stand up within a few seconds, thereby allowing control of the timing of the posture change. None of the TAA patients or HCs had ambulatory difficulties, and all of them possessed full mobility and independence of movement. As such, no differences in the timing or manner of standing were observed by the researchers in any of the cohorts. None of the HCs and TAA patients exhibited presyncope signs during STAND. During the experimental protocol, patients breathed spontaneously and were not allowed to talk. The signals were converted from analog to digital using a commercial device at a sampling rate of 400 Hz (PowerLab, ADInstruments, Australia).

### Variability series extraction

Beat-to-beat variability series of cardiovascular variables were extracted from the ECG, AP, and RESP signals. Each HP was computed from the ECG as the temporal distance between two consecutive R-wave peaks. The *k*th heart rate (HR) was computed as the inverse of the *k*th HP. The *k*th SAP value was defined as the maximum AP value within the *k*th HP. The *k*th diastolic AP (DAP) was defined as the minimum of AP following the *k*th SAP. The *k*th mean AP (MAP) was defined as MAP = (2⸱DAP + SAP)/3 and computed over the *k*th SAP and DAP values. The RESP series was calculated by sampling the RESP signal at the first R-wave peak, delimiting the *k*th HP. The resulting beat-to-beat series were manually checked and corrected in case of missing beats or misdetections (Task Force of the European Society of Cardiology and the North American Society of Pacing and Electrophysiology, 1996). The effects of ectopic beats or isolated arrhythmic events were mitigated via linear interpolation between the closest values unaffected by the arrhythmic beat. Corrections did not exceed 5% of the total sequence length. According to the standards of short-term variability analysis (Task Force of the European Society of Cardiology and the North American Society of Pacing and Electrophysiology, 1996), sequences of 256 consecutive beats were selected at REST and during STAND. The random selection of the onset of the sequence within the session prevented the arbitrary selection of the sequence by the researcher from influencing the analysis. The first 2 minutes during STAND were not considered to avoid transient adjustments of variables due to the posture change.

### Time- and frequency-domain autonomic indexes

In the time domain, we computed the mean (μ) and variance (σ^2^) of HP and SAP beat-to-beat series, and they were labeled as μ_HP_, σ^2^_HP_, μ_SAP_, and σ^2^_SAP_ and expressed in ms, ms^2^, mmHg, and mmHg^2^, respectively. The μ of HR, DAP, and MAP, labeled as μ_HR_, μ_DAP_, and μ_MAP_ and expressed in beats·min^‒1^, mmHg, and mmHg, respectively, were also calculated.

The HP, SAP, and RESP series were analyzed in the frequency domain. The parametric power spectral analysis based on the best fit of the series with a realization of the autoregressive model was performed (Pagani et al., 1986; Task Force of the European Society of Cardiology and the North American Society of Pacing and Electrophysiology, 1996; Baselli et al., 1997). The autoregressive model order was identified between 8 and 14 according to Akaike’s figure of merit (Akaike, 1974), while the model coefficients and the variance of the white noise feeding the autoregressive model were identified by solving the least squares problem via Levinson–Durbin recursion (Kay and Marple, 1981). Spectral components of the HP and SAP series were labeled as low frequency (LF) if their central frequency was between 0.04 and 0.15 Hz and as high frequency (HF) if their central frequency was between 0.15 and 0.4 Hz (Task Force of the European Society of Cardiology and the North American Society of Pacing and Electrophysiology, 1996; Baselli et al., 1997). The sum of the power of the spectral components in each band was taken as the total power in that specific band. The HF power of the HP series (HF_HP_) expressed in ms^2^ was taken as a marker of vagal modulation directed to the sinus node (Pomeranz et al., 1985; La Rovere et al., 2020). The LF power of the SAP series (LF_SAP_) expressed in mmHg^2^ was taken as a marker of sympathetic modulation directed to the vessels (Pagani et al., 1986; Marchi et al., 2016a). The frequency of the dominant oscillation of the RESP series within the HF band was indicated as the respiratory rate (f_RESP_) and expressed in breaths·min^‒1^.

### Baroreflex markers

Baroreflex control markers were computed from the power cross-spectral density function, which was estimated from the SAP and HP series according to a bivariate auto-regressive approach (Porta et al., 2000). The model parameters were identified by solving the least squares problem via the Cholesky decomposition method, and the model order was fixed to 10 (Porta et al., 2000). The transfer function gain is defined as the modulus of the cross-spectral density function from the SAP to HP divided by the power spectral density of the SAP. According to Laude et al. (2004), the transfer function gain from SAP to HP can be taken as baroreflex sensitivity (BRS). BRS was expressed in ms·mmHg^‒1^. The squared coherence (K^2^) function between SAP and HP is defined as the ratio of the square modulus of the cross-spectral density function from SAP to HP to the product of the power spectral densities of SAP and HP. K^2^ represents the strength of the coupling between the SAP and HP series. K^2^ is dimensionless and ranges between 0 (indicating null coupling) and 1 (indicating complete coupling). BRS and K^2^ were sampled in correspondence to the maximum of the K^2^ function in the LF frequency band (Bari et al., 2019), and the values were indicated as BRS_LF_ and K^2^_LF_.

### Statistical analysis

Normality of all variables was assessed using the Shapiro–Wilk test. To assess differences in demographic continuous variables across the three cohorts (*i.e.*, HC, Synd, or NonSynd), one-way analysis of variance, or one-way analysis of variance on ranks when appropriate, was used (Holm–Sidak test for multiple comparisons). The chi-square test was used for categorical variables, and the level of significance was adjusted for the number of comparisons. To evaluate differences in clinical continuous characteristics between Synd and NonSynd patient cohorts, an unpaired Student’s t-test or a Mann–Whitney rank-sum test, when appropriate, was applied. The chi-square test was performed in the presence of categorical variables. Two-way repeated measures analysis of variance (one-factor repetition, Holm–Sidak test for multiple comparisons) was utilized to assess the significance of the differences of cardiovascular and baroreflex indexes across groups (*i.e.*, HC, Synd, or NonSynd) within the same experimental condition (*i.e.*, REST or STAND) and between experimental conditions within the same population. If the null hypothesis of normality was rejected for the variable under analysis, a logarithmic transformation was performed, and the two-way repeated measures analysis of variance was applied on the log-transformed distribution. Linear regression analysis of HF_HP_, LF_SAP_, BRS_LF_, and K^2^_LF_ on aortic diameters was carried out. The Pearson product–moment correlation coefficient *r* and the type-I error probability *p* were computed. Continuous variables were reported as the mean ± standard deviation, while categorical variables were reported as the total number (percentage over the entire cohort). Statistical analysis was performed with a commercial statistical software application (SigmaPlot v.14.0, SYSTAT Software, San Jose, CA, United States). The level of significance was set to 0.05. A type-I error probability p smaller than the level of significance was deemed significant.

## Results

### Population characteristics

Table 1 lists the demographic characteristics of the three cohorts (*i.e.*, HC, Synd, and NonSynd). No statistically significant difference was observed in terms of age, gender distribution, and body mass index.

**TABLE 1:** Demographic characterization of the HC, Synd, and NonSynd groups.

Parameter	HC (n = 28)	Synd (n = 46)	NonSynd (n = 34)
Age [yrs]	43 ± 11	39 ± 12	44 ± 12
Gender [male]	13 (46)	17 (37)	19 (56)
BMI [kg∙m^−2^]	24 ± 4	23 ± 4	23 ± 3

BMI, body mass index. Continuous data are presented as the mean ± standard deviation, while categorical data are presented as the value (percentage).

Table 2 summarizes the main clinical differences between Synd and NonSynd cohorts. As expected, Synd included all MFS and LDS patients, with a high percentage of MFS subjects showing positivity for FBN1 mutations, along with extra-aortic and systemic conditions in both MFS and LDS. Most of the NonSynd group included patients affected by BAV and all patients who presented with familial TAA. The Synd and NonSynd cohorts did not differ for therapy, including the use of BB and ARB. The impact of comorbidities was similar in Synd and NonSynd groups, except for systemic score<7, spinal deformities, dural ectasia, and mitral valve prolapse, which are not considered as possible factors affecting autonomic and cardiovascular control parameters.

**TABLE 2:** Clinical characteristics of the Synd and NonSynd groups.

Category	Parameter	Synd (n = 46)	NonSynd (n = 34)	*p*
Characterization	MFS	38 (76)	0 (0) #	<0.001
	vEDS	4 (9)	3 (9)	ns
	LDS	4 (9)	0 (0)	ns
	BAV	1 (2)	4 (12)	ns
	Familial TAA	0 (0)	19 (56) #	<0.001
	Suspected collagenopathy	0 (0)	1 (3)	ns
	Suspected LDS	1 (2)	2 (6)	ns
	Suspected MFS	1 (2)	4 (12)	ns
	Suspected connective tissue disorder	0 (0)	1 (3)	ns
	Mutation site gene [FBN1]	32 (70)	3 (9) #	<0.001
	Mutation site gene [not FBN1]	7 (15)	10 (29)	ns
	Familiarity	32 (70)	22 (65)	ns
Co-morbidities	Hypertension	10 (22)	10 (29)	ns
	Hyperlipidemia	2 (4)	0 (0)	ns
	Diabetes mellitus	1 (2)	0 (0)	ns
	CVA/TIA	1 (2)	0 (0)	ns
	CKD	0 (0)	1 (3)	ns
	Systemic score <7	17 (37)	28 (82) #	<0.001
	Ocular pathologies	33 (72)	22 (65)	ns
	Ocular surgeries	7 (15)	3 (9)	ns
	Respiratory pathologies	6 (13)	2 (6)	ns
	Thoracic deformities	12 (26)	7 (21)	ns
	Pectus excavatum	2 (4)	5 (15)	ns
	Pectus carinatum	4 (9)	2 (6)	ns
	Thoracic asymmetry	4 (9)	0 (0)	ns
	Spinal deformities	39 (85)	17 (50) #	0.002
	Scoliosis	19 (41)	8 (24)	ns
	Lordosis	0 (0)	0 (0)	ns
	Kyphosis	5 (11)	1 (3)	ns
	Kyphoscoliosis	8 (17)	6 (18)	ns
	Osteoarticular diseases	33 (72)	22 (65)	ns
	Dural ectasia	25 (54)	9 (26) #	0.024
	Mitral valve prolapse	24 (52)	9 (26) #	0.038
Therapy	Beta-blockers	19 (41)	17 (50)	ns
	Angiotensin II receptor blockers	24 (52)	10 (29)	ns
	Diuretics	1 (2)	2 (6)	ns
	Anticoagulants	9 (20)	9 (26)	ns
	Other therapy	16 (35)	18 (53)	ns

MFS, Marfan syndrome; vEDS, vascular Ehlers–Danlos syndrome; LDS, Loeys–Dietz syndrome; BAV, bicuspid aortic valve; TAA, thoracic aortic aneurysm; FBN1, fibrillin-1 gene; CVA/TIA, cerebrovascular accident/transitory ischemic attack; CKD, chronic kidney disease; *p*, type-I error probability; ns: not significant. Categorical data are presented as the value (percentage). The symbol # indicates *p* < 0.05 vs. Synd.

Table 3 presents the echocardiographic parameters of the aortic root and the proximal ascending aorta. Synd and NonSynd populations were significantly different from HC subjects, but their echocardiographic markers were similar.

**TABLE 3:** Echocardiographic measurements in the HC, Synd, and NonSynd groups.

Parameter	HC (n = 28)	Synd (n = 46)	NonSynd (n = 34)	*p*
Aortic valve annulus diameter [mm]	27 ± 5	24 ± 1 *	24 ± 1 *	0.003
Aortic diameter—sinuses of Valsalva [mm]	28 ± 4	38 ± 5 *	37 ± 7 *	<0.001
Aortic diameter—sinotubular junction [mm]	27 ± 4	30 ± 5 *	32 ± 5 *	0.002
Aortic diameter—ascending aorta [mm]	26 ± 3	32 ± 5 *	33 ± 6 *	<0.001

Data are presented as the mean ± standard deviation; *p*, type-I error probability. The symbol * indicates *p* < 0.05 vs. HC.

### Autonomic control and baroreflex indexes

Table 4 shows that, with the expected exception of μ_HP_ and μ_HR_, autonomic control and baroreflex indices did not differ between the BB and noBB subgroups, and this result was the same in both Synd and NonSynd patients. Similarly, in Table 5, no significant difference was detected between the ARB and noARB subgroups in terms of autonomic control or baroreflex indexes in both Synd and NonSynd patients. For this reason, pharmacological treatment was not considered a confounding factor, and the Synd and NonSynd groups merged patients regardless of therapy (*i.e.*, BB and noBB or ARB and noARB).

**TABLE 4:** Time and frequency domain parameters and baroreflex indexes in the BB and noBB subgroups of the TAA cohort.

Parameter	BB (n = 36)		noBB (n = 44)	
	REST	STAND	REST	STAND
μ_HP_ [ms]	957.21 ± 149.52	784.91 ± 137.62*	866.25 ± 129.00^#^	682.19 ± 119.51*,^#^
σ^2^_HP_ [ms^2^]	1695.37 ± 2256.64	1287.49 ± 1299.56	1606.59 ± 1988.45	965.70 ± 997.85
HF_HP_ [ms^2^]	384.39 ± 502.88	184.58 ± 275.43*	438.50 ± 563.33	118.55 ± 159.90*
μ_HR_ [beats·min^‒1^]	64.20 ± 10.16	79.40 ± 14.06*	70.85 ± 11.12^#^	90.57 ± 15.59*,^#^
μ_SAP_ [mmHg]	117.04 ± 17.59	124.79 ± 16.12*	117.45 ± 13.66	125.69 ± 19.07*
σ^2^_SAP_ [mmHg^2^]	28.89 ± 20.64	41.68 ± 20.92*	25.86 ± 15.40	49.10 ± 38.45*
LF_SAP_ [mmHg^2^]	3.41 ± 3.93	13.92 ± 13.57*	3.86 ± 4.27	24.02 ± 34.10*
μ_DAP_ [mmHg]	67.50 ± 12.35	76.68 ± 12.79*	69.80 ± 11.19	77.60 ± 11.46*
μ_MAP_ [mmHg]	84.01 ± 13.28	92.24 ± 12.08*	85.68 ± 10.95	93.67 ± 12.85*
f_RESP_ [breaths·min^‒1^]	18.41 ± 2.20	19.17 ± 1.73	18.95 ± 1.75	18.97 ± 3.13
BRS_LF_ [ms·mmHg^‒1^]	6.17 ± 4.03	4.32 ± 3.27*	7.18 ± 4.76	4.34 ± 3.18*
K^2^_LF_	0.44 ± 0.17	0.58 ± 0.22*	0.51 ± 0.18	0.66 ± 0.16*

REST, at rest in supine position; STAND, during active standing; HP, heart period; HR, heart rate; AP, arterial pressure; SAP, systolic AP; DAP, diastolic AP; MAP, mean AP; μ, mean; σ^2^, variance; μ_HP_, HP mean; μ_HR_, HR mean; σ^2^_HP_, HP variance; LF, low frequency; HF, high frequency; HF_HP_, HF power of the HP series expressed in absolute units; μ_SAP_, SAP mean; μ_DAP_, DAP mean; μ_MAP_, MAP mean; σ^2^_SAP_, SAP variance; LF_SAP_, LF power of the SAP series expressed in absolute units; RESP, thoracic movement signal; f_RESP_, respiratory rate; BRS_LF_, baroreflex sensitivity in LF band; K^2^_LF_, squared coherence in LF band. Data are presented as the mean ± standard deviation. The symbol * indicates *p* < 0.05 vs. REST within the same group (*i.e*., BB or noBB); the symbol # indicates *p* < 0.05 vs. BB within the same experimental condition (*i.e*., REST or STAND).

**TABLE 5:** Time and frequency domain parameters and baroreflex indices in the ARB and noARB subgroups of the TAA cohort.

Parameter	ARB (n = 34)		noARB (n = 46)	
	REST	STAND	REST	STAND
μ_HP_ [ms]	928.38 ± 144.95	734.77 ± 121.78*	890.37 ± 144.07	721.77 ± 147.60*
σ^2^_HP_ [ms^2^]	2034.79 ± 2302.84	1121.82 ± 1279.97*	1372.06 ± 1917.06	1095.72 ± 1051.19
HF_HP_ [ms^2^]	435.78 ± 502.20	165.43 ± 279.24*	400.12 ± 561.90	134.80 ± 166.61*
μ_HR_ [beats·min^‒1^]	66.12 ± 10.02	83.92 ± 14.23*	69.22 ± 11.82	86.50 ± 17.29*
μ_SAP_ [mmHg]	120.51 ± 13.85	126.33 ± 17.23*	115.00 ± 16.15	124.57 ± 18.23*
σ^2^_SAP_ [mmHg^2^]	29.04 ± 19.85	55.48 ± 56.02*	25.88 ± 16.35	47.85 ± 37.65*
LF_SAP_ [mmHg^2^]	3.61 ± 3.65	21.82 ± 30.66*	3.70 ± 4.43	18.04 ± 25.14*
μ_DAP_ [mmHg]	69.16 ± 10.15	77.89 ± 12.97*	68.53 ± 12.77	77.25 ± 11.41*
μ_MAP_ [mmHg]	86.28 ± 10.28	94.03 ± 12.48*	84.02 ± 13.06	93.03 ± 12.47*
f_RESP_ [breaths·min^‒1^]	18.78 ± 2.28	19.44 ± 2.66	18.66 ± 1.73	18.79 ± 2.56
BRS_LF_ [ms·mmHg^‒1^]	6.79 ± 4.50	3.80 ± 2.66*	6.70 ± 4.47	4.70 ± 3.51*
K^2^_LF_	0.49 ± 0.15	0.59 ± 0.20*	0.48 ± 0.20	0.67 ± 0.20*

REST, at rest in supine position; STAND, during active standing; HP, heart period; HR, heart rate; AP, arterial pressure; SAP, systolic AP; DAP, diastolic AP; MAP, mean AP; μ, mean; σ^2^, variance; μ_HP_, HP mean; μ_HR_, HR mean; σ^2^_HP_, HP variance; LF, low frequency; HF, high frequency; HF_HP_, HF power of the HP series expressed in absolute units; μ_SAP_, SAP mean; μ_DAP_, DAP mean; μ_MAP_, MAP mean; σ^2^_SAP_, SAP variance; LF_SAP_, LF power of the SAP series expressed in absolute units; RESP, thoracic movement signal; f_RESP_, respiratory rate; BRS_LF_, baroreflex sensitivity in LF band; K^2^_LF_, squared coherence in LF band. Data are presented as the mean ± standard deviation. The symbol * indicates *p* < 0.05 vs. REST within the same group (*i.e*., ARB or noARB).

Table 6 shows the markers derived from HP, HR, SAP, DAP, MAP, and RESP. Indexes were listed as a function of experimental conditions (*i.e.*, REST and STAND) and populations (*i.e.*, HC, Synd, and NonSynd). According to the expected physiological response, in the TAA patients, STAND induced a statistically significant decrease in μ_HP_ and HF_HP_ and a significant increase in μ_HR_ and LF_SAP_. Furthermore, in the same cohorts, μ_SAP_, μ_DAP_, μ_MAP_, and σ^2^_SAP_ significantly increased in response to STAND as well. In the HC population, STAND induced a significant decrease in μ_HP_ and increases in μ_HR_, σ^2^_SAP_, and LF_SAP_. The expected decrease in HF_HP_ with postural stimulus was observed, but it was not statistically significant in this cohort. μ_SAP,_ μ_DAP_, and μ_MAP_ did not change significantly between the experimental conditions in HC subjects as well. f_RESP_ remained unchanged during STAND in all three experimental groups, but it was significantly higher in the TAA cohorts than in HCs under both experimental conditions. All the autonomic indices remained unchanged in TAA cohorts compared with those in HCs, both at REST and during STAND.

**TABLE 6:** Time and frequency domain parameters in the HC, Synd, and NonSynd groups.

Parameter	HC (n = 28)		Synd (n = 46)		NonSynd (n = 34)	
	REST	STAND	REST	STAND	REST	STAND
μ_HP_ [ms]	890 ± 154	740 ± 114 *	887 ± 158	698 ± 125 *	932 ± 123	767 ± 144 *
σ^2^_HP_ [ms^2^]	1216 ± 871	1441 ± 1232	1672 ± 2043	1129 ± 1145	1609 ± 2197	1076 ± 1158
HF_HP_ [ms^2^]	215 ± 165	94 ± 111	415 ± 518	173 ± 245 *	415 ± 566	113 ± 176 *
μ_HR_ [beats·min^‒1^]	69.65 ± 13.59	82.89 ± 13.10*	69.78 ± 12.50	88.82 ± 16.26*	65.45 ± 8.58	80.85 ± 14.79*
μ_SAP_ [mmHg]	125 ± 17	127 ± 24	116 ± 17	124 ± 19 *	119 ± 14	127 ± 16 *
σ^2^_SAP_ [mmHg^2^]	18.5 ± 13.2	44.2 ± 34.9 *	29.2 ± 20.7	54.5 ± 52.3 *	24.5 ± 12.8	46.3 ± 35.9 *
LF_SAP_ [mmHg^2^]	3.7 ± 3.6	14.7 ± 11.0 *	3.5 ± 4.5	20.2 ± 29.2 *	3.9 ± 3.5	18.7 ± 25.3 *
μ_DAP_ [mmHg]	73.03 ± 10.60	77.45 ± 12.33	67.71 ± 12.83	77.65 ± 13.59*	70.26 ± 9.95	77.33 ± 9.65*
μ_MAP_ [mmHg]	90.19 ± 12.04	94.31 ± 15.30	83.87 ± 13.18	93.02 ± 13.71*	86.41 ± 10.13	93.97 ± 10.94*
f_RESP_ [breaths·min^‒1^]	16.3 ± 2.3	16.7 ± 2.8	18.5 ± 2.2 #	19.1 ± 2.4 #	19.1 ± 2.4 #	19.0 ± 2.8 #

REST, at rest in supine position; STAND, during active standing; HP, heart period; HR, heart rate; AP, arterial pressure; SAP, systolic AP; DAP, diastolic AP; MAP, mean AP; μ = mean; σ^2^, variance; μ_HP_, HP mean; μ_HR_, HR mean; σ^2^_HP_, HP variance; LF, low frequency; HF, high frequency; HF_HP_, HF power of the HP series expressed in absolute units; μ_SAP_, SAP mean; μ_DAP_, DAP mean; μ_MAP_, MAP mean; σ^2^_SAP_, SAP variance; LF_SAP_, LF power of the SAP series expressed in absolute units; RESP, thoracic movement signal; f_RESP_, respiratory rate. Data are presented as the mean ± standard deviation. The symbol * indicates *p* < 0.05 vs. REST within the same group (*i.e*., HC, Synd, or NonSynd); the symbol # indicates *p* < 0.05 vs. HC within the same experimental condition (*i.e*., REST or STAND).

**FIGURE 1:**
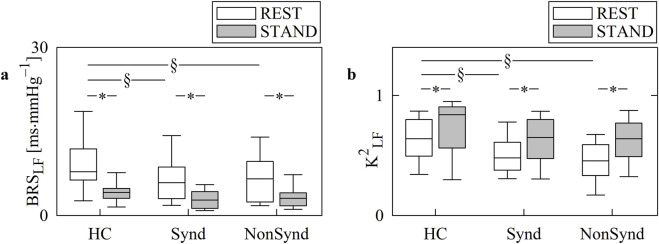
Vertical grouped box-and-whiskers plots show the 5th, 25th, 50th, 75th, and 95th percentiles of BRS_LF_ **(a)** and K^2^_LF_ **(b)** as a function of the experimental groups (*i.e.*, HC, Synd, and NonSynd) at REST (solid white bars) and during STAND (solid gray bars). The height of the box represents the interquartile range, with the median indicated as a horizontal line, while the whiskers correspond to the 5th and 95th percentiles. The symbol * indicates significant variations compared to REST within the experimental groups (*i.e.*, HC, Synd, or NonSynd) with *p* < 0.05. The symbol § indicates significant variations compared to HC within the same experimental condition (*i.e.*, REST or STAND) with *p* < 0.05.

The vertical grouped box-and-whisker plots of Figure 1 show the BRS_LF_ (Figure 1a) and K^2^_LF_ (Figure 1b) as a function of the group (*i.e.*, HC, Synd, and NonSynd) at REST (solid white bars) and during STAND (solid gray bars). In the HC cohort, STAND induced a physiological decrease in BRS_LF_. The reduction in BRS_LF_ during STAND was also detected in both Synd and NonSynd groups. Remarkably, both the TAA groups exhibited lower BRS_LF_ than HCs at REST, while the decrease was not significant during STAND. The expected increase in K^2^_LF_ during STAND was evident in all the groups. Remarkably, Synd and NonSynd patients had significantly lower K^2^_LF_ than HCs during REST, while the decrease was not significant during STAND.

Correlation analysis was carried out separately at REST and during STAND in HC, Synd, and NonSynd groups. None of the markers of autonomic modulation and baroreflex control were significantly associated with any estimate of the aortic diameter.

## Discussion

Our results describe, for the first time, the autonomic control and baroreflex evaluation in Synd and NonSynd cohorts of TAA patients, originally comparing them with HC subjects. The main findings of the present study are as follows: i) the autonomic and baroreflex response to STAND was preserved in both the Synd and NonSynd groups; ii) dampened BRS and strength of HP–SAP coupling were observed in both Synd and NonSynd patients at REST.

### The autonomic and baroreflex response to STAND is preserved in TAA patients

Active standing (STAND) is an orthostatic maneuver known to elicit sympathetic activation and vagal withdrawal (Steptoe and Vögele, 1990; Matsushima et al., 2004; Catai et al., 2014; Milan-Mattos et al., 2018; Porta et al., 2020; Bari et al., 2023; Bari et al., 2024a; Bari et al., 2025). In this work, we observed that autonomic function is preserved in both Synd and NonSynd TAA patients, as indicated by the decrease in μ_HP_ and HF_HP_, suggesting a vagal withdrawal, and by the increased LF_SAP_, supporting an enhanced sympathetic control (Montano et al., 1994; Cooke et al., 1999; Porta et al., 2011; Marchi et al., 2016a). Remarkably, no differences were observed between the two TAA groups in terms of the magnitude of the autonomic response. Compared to HC subjects, TAA patients also showed a similar response to STAND since no significant difference between groups was observed, corroborating the fact that autonomic control is preserved in patients with TAA.

The preservation of the autonomic response to STAND was confirmed by the detection of the expected BRS_LF_ decrease in all groups (Steptoe and Vögele, 1990; Marchi et al., 2016b; De Maria et al., 2019b; De Maria et al., 2023; Bari et al., 2024a), along with an increase in the coupling strength between HP and SAP, as measured by K^2^_LF_ (Milan-Mattos et al., 2018), occurring in all three populations. This result is in agreement with the absence of postural hypotensive/hypertensive episodes reported in our TAA cohorts, indicating a certain level of adaptation of the control systems to the different set points of the Synd and NonSynd groups compared with those in HC subjects.

In the TAA groups, we also observed increased SAP, DAP, and MAP during STAND. This situation could be related to the presence of the aortic aneurysm. We speculate that the aortic aneurysm would further stimulate the baroreceptors, which are already under pressure due to the stretch of the aortic wall. In this situation, even minimal variations in AP gradients, such as those occurring after a postural change, may be undetected by baroreceptors that are already stretched. As a result, they fail to activate compensatory mechanisms to baroreflex unloading. According to this interpretation, any additional stretching of the aortic wall induced by a potential increase of AP could potentially worsen the aneurysm and even produce a further blunting of the baroreflex response, thus favoring a process that might finally lead to the rupture of the aneurysm. The increase of AP during STAND in presence of a physiological baroreflex response of the cardiac arms of the baroreflex is not surprising because it is well known that the degree of engagement of the cardiac arm of the baroreflex has a limited capacity to counteract slow drifts of absolute AP values, whereas it is more effective in limiting SAP variations, especially the positive ones (Steptoe and Vögele, 1990; Rudas et al., 1999; Marchi et al., 2016b; De Maria et al., 2019b; De Maria et al., 2023; Bari et al., 2024b).

### Baroreflex control is dampened in patients with TAA compared to that in HCs

The decrease in BRS_LF_ and the increase in K^2^_LF_ during STAND were expected in HC subjects (Steptoe and Vögele, 1990; Milan-Mattos et al., 2018; De Maria et al., 2019b; Bari et al., 2024b), and a similar impact of STAND was observed in the TAA groups. However, the BRS of HCs was different from that of TAA patients. We observed that HCs exhibited higher BRS values at REST compared to both Synd and NonSynd patients, thus suggesting a depression of baroreflex control in TAA patients. We hypothesize that it may be due to a higher stretch of the aortic baroreflex site in TAA patients related to the presence of the aneurysm, leading to lower BRS_LF_ at REST (Bari et al., 2025). One of the potential reasons leading to this result could also be the greater stiffness of the aortic substrate area close to the location of the mechanoreceptors responsible for initiating the pressure reflex. This situation augmented cardiovascular risk for this population (La Rovere et al., 1998; Ranucci et al., 2017). Furthermore, this difference was observed in both Synd and NonSynd patients, suggesting that the impairment is similar in the two TAA populations and should not be attributed to traits specific to either group; rather, it may be more closely related to common dysfunctions in the mechanical properties of the aortic wall (Bari et al., 2025). Therefore, we can confirm our hypothesis regarding the impact of TAA on baroreflex control but not the hypothesis suggesting a possible difference between Synd and NonSynd groups. Remarkably, this difference compared to HC individuals was not observed during STAND, suggesting that the autonomic nervous system of TAA patients, when appropriately stimulated during STAND (Porta et al., 2020; Bari et al., 2023; Bari et al., 2024a), remains capable of responding to the orthostatic challenge. This observation also supports the preservation of autonomic function, as indicated by spectral markers.

Remarkably, our results represent a first insight into the influence of aortic diameters and arterial wall properties on BRS in TAA groups. Any variation in the aortic diameter and changes in the mechanical properties of the aortic wall may significantly affect the function and sensitivity of baroreceptors (Reutersberg et al., 2022). This might affect the baroreflex function in other pathological conditions, such as hypertension (Lucini et al., 2002), and in patients undergoing surgeries involving baroreceptor sites, such as the aortic arch and the carotid sinus (Porta et al., 2020; Bari et al., 2023; Bari et al., 2024b; Dalla Vecchia et al., 2013). Therefore, these results might clarify possible clinical symptoms related to the aortic dimensions.

Another finding supporting the hypothesis of the involvement of the intrinsic characteristics of the aortic wall in patients with TAA is the absence of difference between the patients with and without regular intake of BBs or ARBs. Although it is a well-known effect that BB therapy increases μ_HP_ by directly suppressing the sympathetic activity of the autonomic nervous system, its effects on the baroreflex are more debated, with variable results depending on the methodology used to evaluate BRS (Parlow et al., 1995; Wesseling et al., 2017). Specifically, methods based on spontaneous HP and SAP fluctuations (Parlow et al., 1995; Wesseling et al., 2017) might not show any significant impact of propranolol in healthy volunteers. This result is in line with our findings comparing TAA patients who underwent BB therapy to those who did not. On the other hand, an interventional method imposing a significant, pharmacologically induced variation in SAP could detect a significant increase in BRS under propranolol administration in healthy subjects (Parlow et al., 1995; Wesseling et al., 2017). Discrepancies between these previous studies and our results may be explained by the method of BRS estimation, dosage, and the type of administration of BB therapy. In our patients, we employed a method based on spontaneous HP and SAP variability, using a lower chronic dosage of BBs compared with that reported in the literature (Parlow et al., 1995; Wesseling et al., 2017). Similarly, ARBs had no impact on our TAA cohort.

This study suggests that both Synd and NonSynd patients showed a reduction in the marker of vagal modulation, such as HF_HP_, during STAND in all groups, but no significant difference was observed between the HF_HP_ power measured in HCs and that in the TAA groups. This finding contrasts with that of a previous study (Cherkas and Zhuraev, 2016) on the cardiac control of patients with FBN1 mutation. Cherkas and Zhuraev (2016) found almost all HP variability indices were decreased compared with those in HCs. This result was attributed to an overactivation of the TGF-β pathway, which could potentially influence HP variability indexes in patients with FBN1 mutations such as MFS due to the dysregulation of different metabolic mechanisms (Cherkas and Zhuraev, 2016). However, in a letter responding to Cherkas and Zhuraev (2016), it was argued that the observed reduction in HP variability markers may be related less to the FBN1 mutation and more to baroreceptor dysfunction resulting from aortic damage (İşcen, 2016). Derangement of the baroreflex regulation of the autonomic vagal outflow could imply a reduction in the magnitude of HP changes (La Rovere et al., 2020). This second hypothesis appears to be more aligned with our results on the baroreflex impairment of TAA groups and more in agreement with our findings on the impact of the aortic root diameter in TAA compared to that in the HC group.

### Linear correlation between cardiovascular control markers and the aortic diameter

The missing correlation between aortic diameters and baroreflex indexes can be attributed to the complexity of baroreflex control, comprising a sensory component, a central neural integration, and efferent neural pathways, while significant correlation might be expected only with measures related to indexes that describe the sensory component solely. No correlation between aortic diameters and autonomic function indexes was observed. We speculate that autonomic markers are even less influenced by the sensory component of baroreflex because of the impact of control mechanisms different from the baroreflex itself.

### Limitations of the study and future developments

We acknowledge that our study has some limitations. We hypothesized that dimensional properties (*i.e.*, diameter) of the aorta at the baroreceptor site affect BRS in TAA patients. However, while aortic diameters as assessed by 2D-transthoracic echocardiography confirmed the presence of an altered aortic root diameter in the TAA cohorts compared to that in the HC group, aortic compliance values were not included in the study. Therefore, any hypothesis involving vascular properties apart from the aortic dimension cannot be completely verified yet. Future studies should implement measurements based on pulse wave velocity to infer arterial stiffness and elasticity and the pulse-pressure method, which uses stroke volume and pressure waveforms, to derive measures of arterial compliance. Moreover, the absence of genetic testing in the HC group precludes the conclusive exclusion of subclinical Synd cases with normal aortic diameter among HC subjects. This study did not control for the menopausal status or the use of hormonal contraceptives. We expect that this lack might have increased the overall dispersion of our data. We acknowledge that the presence of comorbidities might have played a role in determining the differences between the HC and TAA groups. Future studies should aim to control for comorbidities by defining a suitable HC group, thereby limiting the impact of factors that could influence the conclusions beyond aortic diameter. However, it is worth noting that comorbidities were not significantly different between Synd and NonSynd groups in the present study. Finally, we acknowledge that the autonomic response to STAND might be limited, while head-up tilt is a more powerful orthostatic stimulus. Therefore, the use of head-up tilt might induce more evident changes and unveil more subtle differences across groups.

An important limitation of our work is the difference in pharmacological treatment between the HC cohort and the TAA patients. Specifically, a subgroup of Synd and NonSynd patients were undergoing BB therapy at the time of the study enrollment, and no pharmacological washout was performed due to clinical concerns. Despite showing no significant difference between the TAA subgroups under different pharmacological therapies, it cannot be ruled out that our results are partially affected by pharmacological treatment, especially the comparison with HC individuals. Future studies should increase the number of enrolled patients to account for the confounding effects of therapy and further investigate the effect of BB on the baroreflex in TAA.

## Conclusion

This study represents an initial evaluation of the cardiovascular autonomic control and BRS in TAA patients divided into Synd and NonSynd groups compared to an HC population during a postural challenge. Findings showed that the autonomic response to STAND was preserved in both TAA groups and did not substantially differ from that of HCs. On the contrary, baroreflex control appears to be impaired at REST in TAA patients but shows no differences between Synd and NonSynd patients. Our results suggest the potential impact of the aortic diameter and arterial wall properties on baroreflex control in these patients, leading to the hypothesis of a higher cardiovascular risk in TAA patients. These findings pave the way for future studies focusing on mechanical vascular parameters, such as aortic stiffness, which may significantly affect baroreflex function through alterations in mechano-sensitive properties of baroreceptors. Moreover, the absence of significant differences in TAA patients with and without BB therapy additionally stresses a potential key role of the intrinsic characteristics of the aortic wall. Our findings, while confirmed, would improve risk stratification for patients with TAA and might allow the proposal of countermeasures to reduce cardiovascular risk in this population.

## Data Availability

The raw data supporting the conclusions of this article will be made available by the authors, without undue reservation.

## Abbreviations

HP, heart period; HR, heart rate; AP, arterial pressure; SAP, systolic AP; DAP, diastolic AP; MAP, mean AP; RESP, respiratory movement signal; f_RESP_, respiratory rate from RESP signal; TAA, thoracic aortic aneurysm; MFS, Marfan syndrome; LDS, Loeys–Dietz syndrome; EDS, Ehlers–Danlos syndrome; vEDS, vascular Ehlers–Danlos syndrome; BAV, bicuspid aortic valve syndrome; FBN1, fibrillin-1 gene; Synd, syndromic TAA patients; NonSynd, non-syndromic TAA patients; HC, healthy control; ARB, angiotensin receptor blocker; BB, beta blocker; REST, at rest in supine position; STAND, active standing; μ, mean; σ^2^, variance; LF, low frequency; HF, high frequency; BRS, baroreflex sensitivity; K^2^, squared coherence.

## References

[B1] Akaike H. (1974). A new look at the statistical model identification. IEEE Trans. Autom. Control 19, 716–723. 10.1109/TAC.1974.1100705

[B2] Bari V. Vaini E. Pistuddi V. Fantinato A. Cairo B. De Maria B. (2019). Comparison of causal and non-Causal strategies for the assessment of Baroreflex sensitivity in predicting acute kidney dysfunction after coronary artery bypass grafting. Front. Physiol. 10, 1319. 10.3389/fphys.2019.01319 31681021 PMC6813722

[B3] Bari V. Gelpi F. Cairo B. Anguissola M. Pugliese S. De Maria B. (2023). Characterization of cardiovascular and cerebrovascular controls *via* spectral causality analysis in patients undergoing surgical aortic valve replacement during a three-month Follow-Up. Physiol. Meas. 44, 094001. 10.1088/1361-6579/acf992 37703899

[B4] Bari V. Gelpi F. Cairo B. Anguissola M. Acerbi E. Squillace M. (2024a). Impact of surgical aortic valve replacement and transcatheter aortic valve implantation on cardiovascular and cerebrovascular controls: a pilot Study. Physiol. Rep. 12, e70028. 10.14814/phy2.70028 39227321 PMC11371460

[B5] Bari V. Nano G. Baroni I. De Angeli G. Cairo B. Gelpi F. (2024b). Comparison of the impact of carotid endarterectomy and stenting on autonomic and baroreflex regulations: a one-year Follow-up randomized Study. Sci. Rep. 14, 30299. 10.1038/s41598-024-81105-7 39638832 PMC11621527

[B6] Bari V. Cairo B. Gelpi F. Fancoli F. Curcio N. Matrone G. (2025). Joint analysis of cardiovascular control and shear wave elastography to determine carotid plaque vulnerability. J. Clin. Med. 14, 648. 10.3390/jcm14020648 39860656 PMC11766208

[B7] Baselli G. Porta A. Rimoldi O. Pagani M. Cerutti S. (1997). Spectral decomposition in Multichannel recordings based on multivariate parametric identification. IEEE Trans. Biomed. Eng. 44, 1092–1101. 10.1109/10.641336 9353988

[B8] Baumgartner H. Bonhoeffer P. De Groot N. M. de Haan F. Deanfield J. E. Galie N. (2010). Task force on the management of Grown-up congenital heart disease of the European Society of Cardiology (ESC); Association for European Paediatric Cardiology (AEPC); ESC Committee for practice Guidelines (CPG). Eur. Heart J. 31, 2915–2957. 10.1093/eurheartj/ehq249 20801927

[B9] Bruno R. M. Ghiadoni L. Seravalle G. Dell’Oro R. Taddei S. Grassi G. (2012). Sympathetic regulation of vascular function in health and disease. Front. Physiol. 3, 284. 10.3389/FPHYS.2012.00284 22934037 PMC3429057

[B10] Catai A. M. Takahashi A. C. M. Perseguini N. M. Milan J. C. Minatel V. Rehder-Santos P. (2014). Effect of the postural challenge on the dependence of the cardiovascular control complexity on Age. Entropy 16, 6686–6704. 10.3390/E16126686

[B11] Cherkas A. Zhuraev R. A. (2016). Marked decrease in heart rate variability in Marfan Syndrome patients with confirmed FBN1 mutations. Cardiol. J. 23, 23–33. 10.5603/CJ.A2015.0076 26503076

[B12] Compostella L. Compostella C. Russo N. Setzu T. Bellotto F. (2014). Cardiac autonomic dysfunction after aortic surgery. Int. J. Cardiol. 172, e470–e471. 10.1016/J.IJCARD.2014.01.034 24491877

[B13] Compostella L. Russo N. D’Onofrio A. Setzu T. Compostella C. Bottio T. (2015). Abnormal heart rate variability and atrial fibrillation after aortic surgery. Rev. Bras. Cir. Cardiovasc. 30, 55–62. 10.5935/1678-9741.20140100 25859868 PMC4389516

[B14] Cooke W. H. Hoag J. B. Crossman A. A. Kuusela T. A. Tahvanainen K. U. O. Eckberg D. L. (1999). Human responses to upright tilt: a window on central autonomic integration. J. Physiol. 517, 617–628. 10.1111/J.1469-7793.1999.0617T.X 10332107 PMC2269357

[B15] Dalla Vecchia L. Barbic F. Galli A. Pisacreta M. Gornati R. Porretta T. (2013). Favorable effects of carotid endarterectomy on Baroreflex sensitivity and cardiovascular neural modulation: a 4-Month Follow-Up. Am. J. Physiol. Regul. Integr. Comp. Physiol. 304, R1114–R1120. 10.1152/ajpregu.00078.2013 23576607

[B16] de Boer R. W. Karemaker J. M. Strackee J. (1985). Relationships between short-term blood-pressure fluctuations and heart-rate variability in resting subjects. I: a spectral analysis approach. Med. Biol. Eng. Comput. 23, 352–358. 10.1007/BF02441589 4046655

[B17] De Ferrari G. M. Schwartz P. J. (1994). “Autonomic nervous System, myocardial ischemia, and malignant ventricular arrhythmias: experimental findings,” in Myocardial ischemia and arrhythmia. Editors Zehender M. Meinertz T. Just H. (Steinkopff), 233–250. 10.1007/978-3-642-72505-0_16

[B18] De Maria B. Bari V. Cairo B. Vaini E. De Abreu R. M. Perseguini N. M. (2019a). Cardiac baroreflex hysteresis is one of the determinants of the heart period variability asymmetry. Am. J. Physiol. Regul. Integr. Comp. Physiol. 317, R539–R551. 10.1152/ajpregu.00112.2019 31365303

[B19] De Maria B. Bari V. Cairo B. Vaini E. Esler M. Lambert E. (2019b). Characterization of the asymmetry of the cardiac and sympathetic arms of the baroreflex from spontaneous variability during incremental Head-Up tilt. Front. Physiol. 10, 342. 10.3389/fphys.2019.00342 31001137 PMC6454064

[B20] De Maria B. Dalla Vecchia L. A. Bari V. Cairo B. Gelpi F. Perego F. (2023). The degree of engagement of cardiac and sympathetic arms of the baroreflex does not depend on the absolute value and sign of arterial pressure variations. Physiol. Meas. 44, 114002. 10.1088/1361-6579/ad0976 37922536

[B21] Ehlers M. R. Todd R. M. (2017). Genesis and maintenance of attentional biases: the role of the locus Coeruleus-Noradrenaline System. Neural. Plast. 2017, 6817349. 10.1155/2017/6817349 28808590 PMC5541826

[B22] Electrophysiology T. F. A. (1996). Heart rate variability: standards of measurement, physiological interpretation, and clinical use. Circulation 93, 1043–1065. 10.1161/01.CIR.93.5.1043 8598068

[B23] Faivre L. Collod-Beroud G. Loeys B. L. Child A. Binquet C. Gautier E. (2007). Effect of mutation type and location on clinical outcome in 1,013 probands with Marfan Syndrome or related phenotypes and FBN1 mutations: an international Study. Am. J. Hum. Genet. 81, 454–466. 10.1086/520125 17701892 PMC1950837

[B24] Faiza Z. Sharman T. (2025). “Thoracic aorta aneurysm,” in *StatPearls*, treasure Island (FL) (Treasure Island, FL: StatPearls Publishing). Available online at: https://www.ncbi.nlm.nih.gov/books/NBK554567/. 32119454

[B25] Ghitani N. Chesler A. T. (2019). The anatomy of the baroreceptor reflex. Cell. Rep. 29, 2121–2122. 10.1016/J.CELREP.2019.11.031 31747586 PMC8794002

[B26] Hainsworth R. (2014). Cardiovascular control from cardiac and pulmonary vascular receptors. Exp. Physiol. 99, 312–319. 10.1113/EXPPHYSIOL.2013.072637 24058186

[B27] Hoyer D. Friedrich H. Stein P. K. Domitrovich P. P. Hoyer H. Faldu C. (2008). Autonomic information flow improves prognostic value of heart rate patterns after abdominal aortic surgery. J. Crit. Care 23, 255–262. 10.1016/J.JCRC.2007.04.005 18538221

[B28] İşcen S. (2016). Is heart rate variability related to baroreceptors or FBN1 mutations? Cardiol. J. 23, 120. 10.5603/CJ.2016.0013 26927512

[B29] Isselbacher E. M. (2005). Thoracic and abdominal aortic aneurysms. Circulation 111, 816–828. 10.1161/01.CIR.0000154569.08857.7A 15710776

[B30] Kay S. M. Marple S. L. (1981). Spectrum Analysis—A modern perspective. Proc. IEEE 69, 1380–1419. 10.1109/PROC.1981.12184

[B31] Klassen S. A. Chirico D. Dempster K. S. Shoemaker J. K. O’Leary D. D. (2016). Role of aortic arch vascular mechanics in cardiovagal baroreflex sensitivity. Am. J. Physiol. Regul. Integr. Comp. Physiol. 311, R24–R32. 10.1152/ajpregu.00491.2015 27122371

[B32] La Rovere M. T. Bigger Jr J. T. Marcus F. I. Mortara A. Schwartz P. J. (1998). Baroreflex sensitivity and heart-rate variability in prediction of total cardiac mortality after myocardial infarction. ATRAMI (autonomic tone and reflexes after myocardial infarction) investigators. Lancet 351, 478–484. 10.1016/s0140-6736(97)11144-8 9482439

[B33] La Rovere M. T. Porta A. Schwartz P. J. (2020). Autonomic control of the heart and its clinical impact. A personal perspective. Front. Physiol. 11, 582. 10.3389/fphys.2020.00582 32670079 PMC7328903

[B34] Laude D. Elghozi J. L. Girard A. Bellard E. Bouhaddi M. Castiglioni P. (2004). Comparison of various techniques used to estimate spontaneous Baroreflex Sensitivity (the EuroBaVar Study). Am. J. Physiol. Regul. Integr. Comp. Physiol. 286, R226–R231. 10.1152/AJPREGU.00709.2002 14500269

[B35] Loeys B. L. Schwarze U. Holm T. Callewaert B. L. Thomas G. H. Pannu H. (2006). Aneurysm syndromes caused by mutations in the TGF-Beta receptor. N. Engl. J. Med. 355, 788–798. 10.1056/NEJMOA055695 16928994

[B36] Loeys B. L. Dietz H. C. Braverman A. C. Callewaert B. L. De Backer J. Devereux R. B. (2010). The revised ghent nosology for the Marfan Syndrome. J. Med. Genet. 47, 476–485. 10.1136/JMG.2009.072785 20591885

[B37] Lucini D. Mela G. S. Malliani A. Pagani M. (2002). Impairment in cardiac autonomic regulation preceding arterial hypertension in humans: insights from spectral analysis of beat-by-beat cardiovascular variability. Circulation 106, 2673–2679. 10.1161/01.cir.0000039106.89299.ab 12438292

[B38] MacCarrick G. Black J. H. Bowdin S. El-Hamamsy I. Frischmeyer-Guerrerio P. A. Guerrerio A. L. (2014). Loeys-Dietz syndrome: a primer for diagnosis and management. Genet. Med. 16, 576–587. 10.1038/GIM.2014.11 24577266 PMC4131122

[B39] Malfait F. Francomano C. Byers P. Belmont J. Berglund B. Black J. (2017). The 2017 international classification of the ehlers-danlos syndromes. Am. J. Med. Genet. C Semin. Med. Genet. 175, 8–26. 10.1002/AJMG.C.31552 28306229

[B40] Marchi A. Bari V. De Maria B. Esler M. Lambert E. Baumert M. (2016a). Calibrated variability of muscle sympathetic nerve activity during graded head-up tilt in humans and its link with noradrenaline data and cardiovascular rhythms. Am. J. Physiol. Regul. Integr. Comp. Physiol. 310, R1134–R1143. 10.1152/ajpregu.00541.2015 27009053

[B41] Marchi A. Bari V. De Maria B. Esler M. Lambert E. Baumert M. (2016b). Simultaneous characterization of sympathetic and cardiac arms of the baroreflex through sequence techniques during incremental Head-Up tilt. Front. Physiol. 7, 438. 10.3389/fphys.2016.00438 27746741 PMC5041323

[B42] Marelli S. Micaglio E. Taurino J. Salvi P. Rurali E. Perrucci G. L. (2023). Marfan Syndrome: enhanced diagnostic tools and Follow-up management strategies. Diagnostics 13, 2284. 10.3390/diagnostics13132284 37443678 PMC10340634

[B43] Matsushima R. Tanaka H. Tamai H. (2004). Comparison of the active standing Test and Head-up tilt Test for diagnosis of syncope in childhood and adolescence. Clin. Auton. Res. 14, 376–384. 10.1007/s10286-004-0182-2 15666065

[B44] Milan-Mattos J. C. Porta A. Perseguini N. M. Minatel V. Rehder-Santos P. Takahashi A. C. M. (2018). Influence of age and gender on the phase and strength of the relation between heart period and systolic blood pressure spontaneous fluctuations. J. Appl. Physiol. 124, 791–804. 10.1152/japplphysiol.00903.2017 29212671

[B45] Montano N. Ruscone T. G. Porta A. Lombardi F. Pagani M. Malliani A. (1994). Power spectrum analysis of heart rate variability to assess the changes in sympathovagal balance during graded orthostatic tilt. Circulation 90, 1826–1831. 10.1161/01.cir.90.4.1826 7923668

[B46] Pagani M. Lombardi F. Guzzetti S. Rimoldi O. Furlan R. Pizzinelli P. (1986). Power spectral analysis of heart rate and arterial pressure variabilities as a marker of sympatho-vagal interaction in man and conscious dog. Circ. Res. 59, 178–193. 10.1161/01.RES.59.2.178 2874900

[B47] Parati G. Di Rienzo M. Bertinieri G. Pomidossi G. Casadei R. Groppelli A. (1988). Evaluation of the baroreceptor-heart rate reflex by 24-Hour intra-arterial blood pressure monitoring in humans. Hypertension 12, 214–222. 10.1161/01.HYP.12.2.214 3410530

[B48] Parlow J. Viale J. P. Annat G. Hughson R. Quintin L. (1995). Spontaneous Cardiac baroreflex in humans: Comparison with drug-induced responses. Hypertension 25, 1058–1068. 10.1161/01.HYP.25.5.1058 7737717

[B49] Pitcher A. Spata E. Emberson J. Davies K. Halls H. Holland L. (2022). Angiotensin receptor blockers and β blockers in Marfan Syndrome: an individual patient data meta-analysis of randomised trials. Lancet 400, 822–831. 10.1016/S0140-6736(22)01534-3 36049495 PMC7613630

[B50] Pomeranz B. Macaulay J. B. Caudill M. A. Kutz I. Adam D. Gordon D. (1985). Assessment of autonomic functions in humans by heart rate spectral analysis. Am. J. Physiol. Heart Circ. Physiol. 17, H151–H153. 10.1152/ajpheart.1985.248.1.h151 3970172

[B51] Porta A. Baselli G. Rimoldi O. Malliani A. Pagani M. (2000). Assessing baroreflex gain from spontaneous variability in conscious dogs: role of causality and respiration. *Am. J. Physiol.* Heart Circ. Physiol. 279, H2558–H2567. 10.1152/ajpheart.2000.279.5.h2558 11045994

[B52] Porta A. Furlan R. Rimoldi O. Pagani M. Malliani A. Van De Borne P. (2002). Quantifying the strength of the linear causal coupling in closed loop interacting cardiovascular variability signals. Biol. Cybern. 86, 241–251. 10.1007/S00422-001-0292-Z/METRICS 12068789

[B53] Porta A. Bari V. Badilini F. Tobaldini E. Gnecchi-Ruscone T. Montano N. (2011). Frequency domain assessment of the coupling strength between ventricular repolarization duration and heart period during graded Head-up tilt. J. Electrocardiol. 44, 662–668. 10.1016/j.jelectrocard.2011.08.002 21908003 PMC3206996

[B54] Porta A. Bari V. Bassani T. Marchi A. Pistuddi V. Ranucci M. (2013). Model-based causal closed-loop approach to the estimate of baroreflex sensitivity during propofol anesthesia in patients undergoing coronary artery bypass graft. J. Appl. Physiol. 115, 1032–1042. 10.1152/japplphysiol.00537.2013 23869064

[B55] Porta A. Fantinato A. Bari V. Gelpi F. Cairo B. De Maria B. (2020). Evaluation of the impact of surgical aortic valve replacement on short-term cardiovascular and cerebrovascular controls through spontaneous variability analysis. PLoS ONE 15, e0243869. 10.1371/journal.pone.0243869 33301491 PMC7728248

[B56] Ranucci M. Porta A. Bari V. Pistuddi V. La Rovere M. T. (2017). Baroreflex sensitivity and outcomes following coronary surgery. PLoS ONE 12, e0175008. 10.1371/journal.pone.0175008 28384188 PMC5383149

[B57] Reutersberg B. Pelisek J. Ouda A. de Rougemont O. Rössler F. Zimmermann A. (2022). Baroreceptors in the aortic arch and their potential role in aortic dissection and aneurysms. J. Clin. Med. 11, 1161. 10.3390/JCM11051161 35268252 PMC8911340

[B58] Robinson P. N. Arteaga-Solis E. Baldock C. Collod-Béroud G. Booms P. De Paepe A. (2006). The molecular genetics of Marfan Syndrome and related disorders. J. Med. Genet. 43, 769–787. 10.1136/JMG.2005.039669 16571647 PMC2563177

[B59] Rudas L. Crossman A. A. Morillo C. A. Halliwill J. R. Tahvanainen K. U. O. Kuusela T. A. (1999). Human sympathetic and vagal baroreflex responses to sequential nitroprusside and phenylephrine. Am. J. Physiol. 276, H1691–H1698. 10.1152/ajpheart.1999.276.5.h1691 10330255

[B60] Stein P. K. Schmieg R. E. El-Fouly A. Domitrovich P. P. Buchman T. G. (2001). Association between heart rate variability recorded on postoperative day 1 and length of stay in abdominal aortic surgery patients. Crit. Care Med. 29, 1738–1743. 10.1097/00003246-200109000-00014 11546974

[B61] Steptoe A. Vögele C. (1990). Cardiac baroreflex function during postural change assessed using non-invasive spontaneous sequence analysis in young men. Cardiovasc. Res. 24, 627–632. 10.1093/CVR/24.8.627 2224929

[B62] Wesseling K. H. Karemaker J. M. Castiglioni P. Toader E. Cividjian A. Settels J. J. (2017). Validity and variability of XBRS: instantaneous Cardiac baroreflex sensitivity. Physiol. Rep. 5, e13509. 10.14814/PHY2.13509 29180481 PMC5704083

